# Neuroimaging the traumatized self: fMRI reveals altered response in cortical midline structures and occipital cortex during visual and verbal self- and other-referential processing in women with PTSD

**DOI:** 10.1080/20008198.2017.1314164

**Published:** 2017-05-16

**Authors:** Paul Frewen, Elizabeth Thornley, Daniela Rabellino, Ruth Lanius

**Affiliations:** ^a^ School and Applied Child Psychology, University of Western Ontario, London, ON, Canada; ^b^ Posttraumatic Stress Disorder (PTSD) Research Unit, University of Western Ontario, London, ON, Canada

**Keywords:** Self, trauma, Posttraumatic Stress Disorder, PTSD, fMRI, intrinsic connectivity networks

## Abstract

**Background**: Changes to the diagnostic criteria for PTSD in DSM-5 reflect an increased emphasis on negative cognition referring to self and other, including self-blame, and related pervasive negative affective states including for self-conscious emotions such as guilt and shame.

**Objective**: Investigate the neural correlates of valenced self-referential processing (SRP) and other-referential processing (ORP) in persons with PTSD.

**Method**: We compared response to the Visual-Verbal Self-Other Referential Processing Task in an fMRI study of women with (n = 20) versus without (n = 24) PTSD primarily relating to childhood and interpersonal trauma histories using statistical parametric mapping and group independent component analysis.

**Results**: As compared to women without PTSD, women with PTSD endorsed negative words as more descriptive both of themselves and others, whereas positive words were endorsed as less descriptive both of themselves and others. Women with PTSD also reported a greater experience of negative affect and a lesser experience of positive affect during SRP specifically. Significant differences between groups were observed within independent components defined by ventral- and middle-medial prefrontal corte x, mediolateral parietal cortex, and visual cortex, depending on experimental conditions.

**Conclusions**: This study reveals brain-based disturbances during SRP and ORP in women with PTSD related to interpersonal and developmental trauma. Psychological assessment and treatment should address altered sense of self and affective response to others in PTSD.

Whereas in recent history Posttraumatic Stress Disorder (PTSD) was primarily regarded as a fear-based anxiety disorder primarily associated with the processing of external threat, diagnostic practices now also focus on negative self-referential and other-referential cognitive appraisals that are also frequently present in persons with PTSD, including persistent and exaggerated negative expectations for oneself, as well as a distorted sense of self- or other-focused blame for the causes and consequences of the traumatic event (American Psychiatric Association [APA], 2013; Cox, Resnick, & Kilpatrick, 2014; Friedman, Resick, Bryant, & Brewin, 2011). Moreover, current diagnostic practices also emphasize that persons with PTSD often experience pervasive negative emotional states, including for self-conscious, internally-directed emotions like guilt and shame (e.g. Budden, 2009; Dorahy et al., 2015; Dyer et al., 2017; Frewen, Schmittmann, Bringmann, & Borsboom, 2013; Herman, 2011; Lee, Scragg, & Turner, 2010; Stotz, Elbert, Müller, & Schauer, 2015; Taylor, 2015).

Neuroimaging studies reveal brain regions that are often engaged by tasks that explicitly assess participants’ way of thinking and feeling about themselves, including cortical midline structures such as the ventromedial prefrontal cortex, the perigenual anterior cingulate cortex, the dorsomedial prefrontal cortex, the medial parietal cortex or precuneus, the posterior cingulate cortex, and the retrosplenial cortex (Denny, Kober, Wager, & Ochsner, 2012; Gillihan & Farah, 2005; Northoff & Bermpohl, 2004; Northoff et al., 2006; Qin & Northoff, 2011; Van Overwalle, 2011). As such, brain regions activated during self-referential processing (SRP) overlap with what has been termed the default-mode network, one of the primary intrinsic connectivity networks thought to be most active during internally-focused thought and autobiographical memory (Qin & Northoff, 2011; Spreng, Mar, & Kim, 2009; Toro, Fox, & Paus, 2008). However, whereas cortical midline structures are often regarded as responding as an intrinsic connectivity network, cluster and factor analyses reveal independent activation foci during performance of SRP tasks, for example, differentiating between response within ventral and dorsal medial prefrontal cortex, as well as between anterior and posterior regions such as the posterior cingulate and precuneus (Denny et al., 2012; Northoff et al., 2006). Moreover, these brain regions reveal functional specificity during SRP tasks such that, for example, although earlier analyses only showed overlapping response within medial prefrontal cortex when participants thought about themselves (SRP) and when participants were required to think about others (i.e. other-referential processing [ORP]; Gillihan & Farah, 2005; Qin & Northoff, 2011; Van Overwalle, 2011), more recent analyses suggest that SRP is more strongly associated with response within ventral medial prefrontal cortex (BA10) and the perigenual anterior cingulate cortex (BA32), whereas ORP may be more strongly associated with dorsal medial prefrontal cortex (BA8 and BA9) along a dorsal-ventral response gradient (Denny et al., 2012; Murray, Schaer, & Debbané, 2012). Additionally, meta-analysis reveals an anterior-posterior dissociation further suggestive of SRP–ORP specificity, respectively (Araujo, Kaplan, & Damasio, 2013). Finally, studies suggest that it is response within the middle medial prefrontal cortex that most overlaps that seen during resting state (D’Argembeau et al., 2005; Whitfield-Gabrieli et al., 2011). Interestingly, neuroimaging studies in PTSD also reliably implicate altered connectivity within the default-mode network (Bluhm et al., 2009; Cisler, Steele, Smitherman, Lenow, & Kilts, 2013; Sripada et al., 2012; Tursich et al., 2015; Zhang et al., 2015) as well as within other intrinsic connectivity networks including the salience network (Cisler et al., 2013; Rabellino et al., 2015; Sripada et al., 2012; Tursich et al., 2015; Zhang et al., 2015), which includes hubs within the mid-dorsal cingulate cortex, insula, and amygdala and is involved in the detection and subsequent orienting toward important environmental stimuli (Cauda et al., 2011; Dosenbach et al., 2007; Seeley et al., 2007; Sridharan, Levitin, & Menon,).

In addition to investigations of response within brain regions associated with the default mode network and salience network, correlated response within visual cortex is among the most consistently identified independent components in neuroimaging (e.g. Moussa, Steen, Laurienti, & Hayasaka, 2012) and its role in SRP and ORP is increasingly being considered. Indeed, a number of previous studies observed increased response during self-face vs. control conditions in occipital cortex (e.g. for the right inferior occipital gyrus; Kaplan, Aziz-Zadeh, Uddin, & Iacoboni, 2008; Uddin, Kaplan, Molnar-Szakacs, Zaidel, & Iacoboni, 2005). Moreover, enhanced visual processing of emotional relative to neutral pictures (Phan et al., 2002), including specifically faces (Fusar-Poli et al., 2009) has long been known to occur in human functional neuroimaging, where specific visual cortical regions are known to mediate identification and recognition of complex visual arrays such as faces and body parts (Spiridon, Fischl, & Kanwisher, 2006). Moreover, a recent study involving visual processing of emotional pictures found that pictures judged as self-referential not only invoked greater response within both the anterior and posterior cingulate and medial prefrontal cortex, but also preferentially activated the occipital cortex including for the fusiform face area (Herold, Spengler, Sajonz, Usnich, & Bermpohl, 2016). In addition, employing a standard adjective-rating task in women with borderline personality disorder relative to controls, less response within occipital cortex (specifically the fusiform gyrus, lateral occipital lobe), coupled with increased response within both anterior and posterior cortical midline structures, was observed across both SRP and ORP of relatively neutral personality traits (Beeney, Hallquist, Ellison, & Levy, 2016). Beeny and colleagues further found that such effects were associated with behavioural and self-report measures of inconsistency in self and other representation, leading the authors to interpret their findings as potentially reflecting excessive attempts at understanding self in relation to others that may be less grounded in sensory (e.g. visual) information in persons with borderline personality disorder (BPD).

To our knowledge, only two neuroimaging studies have directly investigated SRP in persons with PTSD to date (Frewen et al., 2011; Bluhm et al., 2012). Limitations of both studies, however, include the exclusive focus on SRP to the neglect of its relationship with ORP, a question of longstanding significance within the literature. Moreover, both studies only utilized region-of-interest approaches to analysis, whereas neuroimaging studies increasingly recognize SRP and ORP to be mediated by complex neural networks comprised of multiple functionally-connected hubs as potentially identified by independent component analysis.

We therefore undertook a neuroimaging study comparing response in women with versus without PTSD to the Visual-Verbal Self-Other Referential Processing Task (VV-SORP-T), a novel experimental paradigm that, as its name implies, requires both SRP and ORP of positive and negative stimuli in both the visual and verbal modalities (Frewen & Lundberg, 2012; Frewen, Lundberg, Brimson-Theberge, & Theberge, 2013). In analyses of self-reported data, we hypothesized to observe greater endorsement of negative adjectives and greater negative affect in response to SRP and ORP in persons with PTSD, the opposite being true of positive adjective endorsement and positive affect. In addition, taking into account the integrated visual and verbal nature of the VV-SORP-T and its previously described affective salience, activation differences were hypothesized between healthy women and women with PTSD within neural network hubs identified by independent component analysis and focused on the default mode network (cortical midline structures), the salience network, and visual processing (occipital cortex).

## 1. Method

### 1.1 Participants

Twenty right-handed female participants with a primary diagnosis of PTSD and 24 right-handed female healthy participants were included in the study, all recruited through community advertisements. Exclusion criteria for the PTSD group included substance or alcohol use disorder in the last six months (abuse or dependence), a history of psychosis, bipolar disorder, significant medical conditions, significant head injury, neurologic illness, and fMRI incompatibility (e.g. magnetic or electronic implant), and use of psychotropic medication for at least six weeks before scanning. Exclusion criteria for the control group were the same as for the PTSD group, but also included any lifetime psychiatric disorder. Trauma exposure was neither an inclusionary or exclusionary criterion for the control group.

### 1.2 Materials

#### 1.2.1 Visual-verbal self-other referential processing task

**Figure 1.**
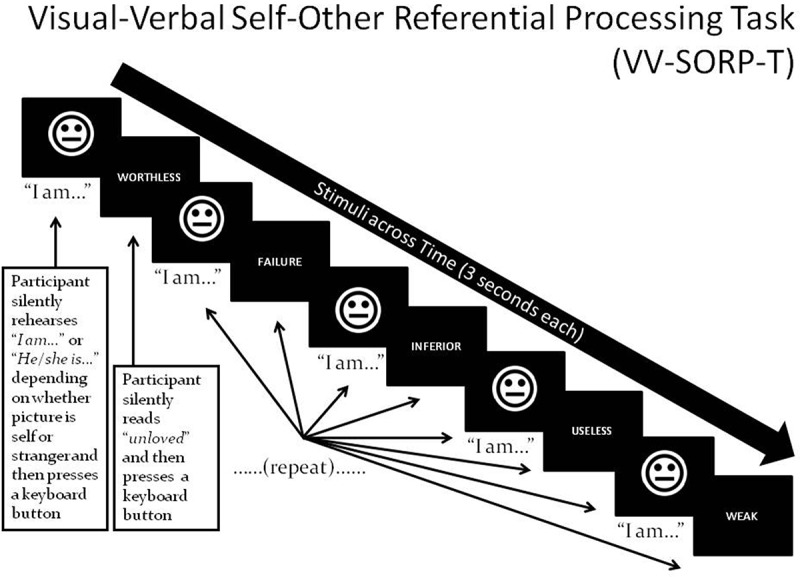
Stimuli are photos of participant (‘Self’) or a stranger (‘Other’) in neutral expression, and positive/negative words. A single block involves presenting five face–word pairings as shown, preceded by a fixation cross (+) for 12 s and the capitalized word ‘SELF’ or ‘OTHER’ for 3 s, depending on whether their own or the stranger’s picture will be presented in the upcoming block. Participants are asked to silently rehearse the statements ‘I am’ or ‘She is’ when they view the Self and Other faces, respectively, and read the words, thus associating the self/other with positivity or negativity. Participants press a response button after silently rehearsing each statement and their reaction time is recorded.

Figure 1 illustrates an example trial of the VV-SORP-T (Frewen & Lundberg, 2012; Frewen, Lundberg, Brimson-Théberge, & Théberge, 2013). The VV-SORP-T involved presenting neutral faces (of the participant herself, or of a stranger) and words (positive and negative) for 3 s in blocks of five face–word pairings. Note that photographs of participants were taken in neutral expression with the instruction to pose as if for a passport photograph, while the non-self faces were collected from the NimStim set of neutral facial expressions (Tottenham et al., 2009) as matched to the self-faces by gender, ethnicity, hair colour, and approximate hair length. All participants underwent three 6-min fMRI runs each including eight blocks. Each block consisted in an initial fixation cross (12 s) followed by the instruction ‘SELF’ or ‘OTHER’ (3 s). The participant’s vs. stranger’s photograph followed for 3 s, during which participants had to silently rehearse to themselves ‘I am’ or ‘She is’, respectively, and then press a keypad button with either their index or middle finger (counter-balanced). They were then presented with a positive or negative word (3 s), asked to silently read the word and then press another keypad button with their other finger. This process was then repeated for four additional photographs and words. In summary, for each block, five pictures and five words were presented following the same ‘picture-then-word’ rotation, with the identical picture displayed in all cases, and the words being of common valence. The stimulus presentations were thereby blocked in terms of the conditions *Reference* (Self vs Other, i.e. photographs) and *Valence* (words), creating four trial types: self-negative, self-positive, other-negative, and other-positive. The order of the eight blocks was randomized within runs, as well as within and across participants. To control for picture novelty, participants were habituated to all photos for 6-10 s prior to administration of the task proper. The button press was intended to confirm attention to the task, although reaction times have been previously interpreted to indicate depth of reflective processing (Frewen & Lundberg, 2012). The same previous validation studies found that, in undergraduates, reaction times were generally slower for SRP trials vs. ORP trials, and for trials involving positive vs. negative words, and that such reaction times correlated with valence congruent adjective endorsement and affective responses to the task.

Before the task, participants also rated how well each word described how they think about themselves, and how well it described how they think about others, on an 11-point scale (0–10) with (0) referring to ‘Not at all’, (5) being ‘Moderately’, and (10) being ‘Completely’. In comparison, after the task, participants rated how they felt emotionally during each of the four trial types relative to standard positive (‘happy’ and ‘good about self’) and negative (‘anger’, ‘sadness’, ‘anxiety-fear’, ‘disgust’, and ‘bad about self’) emotion adjectives using a percentage rating scale with 0% referring to ‘Not at all’, 50% indicating ‘Moderately’, and 100% indicating ‘Strongly’. The adjectives presented reflected social and achievement-based themes, and endorsement rates correlated with responses to the *Rosenberg Self-Esteem Scale* in a prior study (*r* = .73; Frewen & Lundberg, 2012). Moreover, the positive and negative word lists were statistically comparable in terms of length in letters, frequency of use within the English language relative Hyperspace Analogue of Language (HAL) norms (Lund & Burgess, 1996), and in arousal rating relative to the Affective Norms for English Words (Bradley & Lang, 1999) as reported by Frewen, Lundberg, et al., (2013).

### 1.3 Symptom inventories

In brief, the Structured Clinical Interview for DSM-IV Axis I Disorders (SCID-I; First, Spitzer, Gibbon, & Williams, 2002) was administered by trained psychologists in order to assess the presence of current and lifetime psychiatric disorders, excepting the PTSD section. In place of the SCID-I PTSD section, we administered the Clinician Administered PTSD Scale (CAPS; with a cut-off score of >50; Blake et al., 1995), which assesses the frequency and severity of each of the DSM-IV PTSD symptoms. In order to assess childhood trauma history, we administered the Childhood Trauma Questionnaire (CTQ), a 28-item self-report measure of childhood exposure to emotional abuse, emotional neglect, physical abuse, physical neglect, and sexual abuse (Bernstein et al., 2003). Further, in order to obtain a standard, self-report measure of trauma-related negative cognitions referring to oneself, the world, and to self-blame, we administered the Posttraumatic Cognitions Inventory (PTCI; Foa, Ehlers, Clark, Tolin, & Orsillo, 1999).

### 1.4 Procedure

The study was approved by the Health Sciences Research Ethics Board of Western University, Canada, and informed written consent was obtained from all participants. Participants were assessed for inclusion criteria and completed structured clinical diagnostic interviews for PTSD (CAPS), comorbid psychological disorders (SCID-I), and related questionnaires (CTQ, PTCI) approximately two weeks before their scanning date. On the day of scanning, participants first completed a single-block practice version of the VV-SORP-T in an office setting, followed by a resting scan and three blocks of the experimental paradigm while undergoing fMRI (data acquisition and preprocessing specifications are described in Supplemental data). Immediately after scanning, participants completed the affective response rating. The length of the experiment was approximately 75 minutes.

### 1.5 Statistical analyses

#### 1.5.1 Demographics and psychological characteristics

Independent sample *t*-tests were performed to investigate between-group differences for age, CAPS, CTQ, and PTCI scores.

#### 1.5.2 Self-report and behavioural (reaction time) response to the VV-SORP-T

Split-plot MANOVA was performed with adjective endorsement, negative affect ratings, positive affect ratings, and reaction time serving as the dependent measures; MANOVA crossed the within-subject factors *Reference* (SRP vs. ORP) and *Valence* (Negative vs. Positive) with the between-subjects factor *Group* (PTSD vs. Control).

#### 1.5.3 fMRI functional connectivity analysis

##### 1.5.3.1 Independent-component analysis (ICA)

Group ICA was performed using the GIFT fMRI toolbox (GIFT v1.3i; Calhoun, Adali, Pearlson, & Pekar, 2001a) implemented in MATLAB. Group ICA of fMRI data is a multivariate data-driven technique to identify brain areas that are functionally coupled during a task and that are defined as intrinsic connectivity networks (Calhoun, Liu, & Adali, 2009). Spatio-temporal structure contained in the data was obtained via extraction of statistically independent time courses (McKeown & Sejnowski, 1998). One spatial group ICA was performed across all participants and conditions in order to capture both inter-subject and inter-group differences in independent component (IC) spatial extent and amplitude (Allen, Erhardt, Wei, Eichele, & Calhoun, 2012). According to minimum description length criteria, 20 ICs were extracted using the Infomax algorithm (Rosazza, Minati, Ghielmetti, Mandelli, & Bruzzone, 2012), then 20 repetitions of the estimation were run through ICASSO to ensure the reliability of the components (Erhardt et al., 2011; Himberg, Hyvärinen, & Esposito, 2004). The obtained set of averaged group components was then back-reconstructed using principal component analysis compression and projection (GICA1 back-reconstruction method; Calhoun et al., 2001a) into individual spatial maps and time courses and converted into *z-*scores, which represent the contribution of each voxel to the component’s time course (Erhardt et al., 2011).

##### 1.5.3.2 Component selection

The obtained components were firstly visually inspected to reject ICs of obvious artefact (i.e. edges, ventricles, white matter, and artefact signals). A multiple regression using the temporal sorting function in GIFT then allowed us to compare the study model’s time course with the IC time courses in order to identify the ICs most related to the experimental design. For this purpose, we implemented a first level design matrix specifying all conditions as regressors and previously used in a published study (Frewen, Lundberg et al., 2013). Accordingly, a final set of 6 artefact-free task-related ICs represented our focus in this study. All ICs were visually identified with reference to Montemurro and colleagues’ manual (Montemurro & Bruni, 1988), anatomically labelled using the xjView MATLAB toolbox (http://www.alivelearn.net/xjview), and visualized through MRIcron (http://www.mccauslandcenter.sc.edu/mricro/mricron). Furthermore, the spatial maps of the obtained ICs were spatially sorted in GIFT in order to investigate their correlation with templates of recognized networks, specifically the default mode network, the salience network, and the visual network (Garrity et al., 2007; Ros et al., 2013; Shirer, Ryali, Rykhlevskaia, Menon, & Greicius, 2012).

##### 1.5.3.3 Temporal comparison

A multiple regression analysis performed using the temporal sorting function in GIFT resulted in beta weights for each subject, session and condition, which were then used to perform one-way ANOVAs to compare the task-relatedness of each component in the PTSD vs. healthy control groups. We first determined the significance of group comparisons for the SRP and ORP conditions overall, and only in the presence of significant effects were the results examined further for the four valence-specific experimental conditions.

## 2. Results

Table 1 reports descriptive statistics referring to clinical and demographic info. The PTSD group scored higher on the CAPS (*p* < .001) and all CTQ (all *p* < .001) and PTCI (all *p* < .001) subscales. The ethnic origin of the two samples was evenly distributed, although the PTSD group was somewhat older than the control group.

**Table 1.** Demographic and psychological characteristics.

Demographic and psychological characteristics	PTSD group (*n *= 20)	Controls (*n *= 24)
Age (mean ± *SD*) years	35.05 ± 12.64	27.37 ± 7.87
Ethnicity (*n* (group))	16 (EC), 2 (A), 1 (ME)	15 (EC), 6 (A), 2 (AF), 1 (ME)
CAPS score (mean ± SD)	72.55 ± 17.14	0.38 ± 1.36
CTQ emotional abuse score (mean ± *SD*)	17.39 ± 6.78	6.59 ± 3.36
CTQ physical abuse score (mean ± *SD*)	11.95 ± 5.87	5.68 ± 2.15
CTQ sexual abuse score (mean ± *SD*)	14.45 ± 6.18	5.23 ± 1.06
CTQ emotional neglect score (mean ± *SD*)	16.4 ± 5.92	8.82 ± 4.47
CTQ physical neglect score (mean ± *SD*)	11.7 ± 4.80	6.45 ± 2.95
PTCI self (mean ± *SD*)	82.25 ± 29.15	26.5 ± 7.22
PTCI world (mean ± *SD*)	37.44 ± 9.63	16.43 ± 7.94
PTCI shame (mean ± *SD*)	20.56 ± 6.95	7.28 ± 4.76
AXIS I comorbidity (current [past] frequency)	Major depressive disorder (8 [5])	
	Dysthymic disorder (0 [3])	
	Panic disorder with agoraphobia (0[3])	
	Panic disorder without agoraphobia (4[0])	
	Agoraphobia without panic disorder (2[0])	
	Social phobia (4[0])	
	Specific phobia (2[0])	
	Obsessive-compulsive disorder (0[1])	
	Generalized anxiety disorder (1[0])	
	Eating disorders (0[4])	
	Somatoform disorder (9[3])	
	Lifetime history of alcohol abuse or dependence [7]	
	Lifetime history of substance abuse or dependence [7]	

**Legend**: A = Asian, AF = African, EC = European Caucasian, ME = Middle Eastern, PTSD = Posttraumatic Stress Disorder, SD = standard deviation.

### 2.1 Self-report and behavioural results

**Figure 2.**
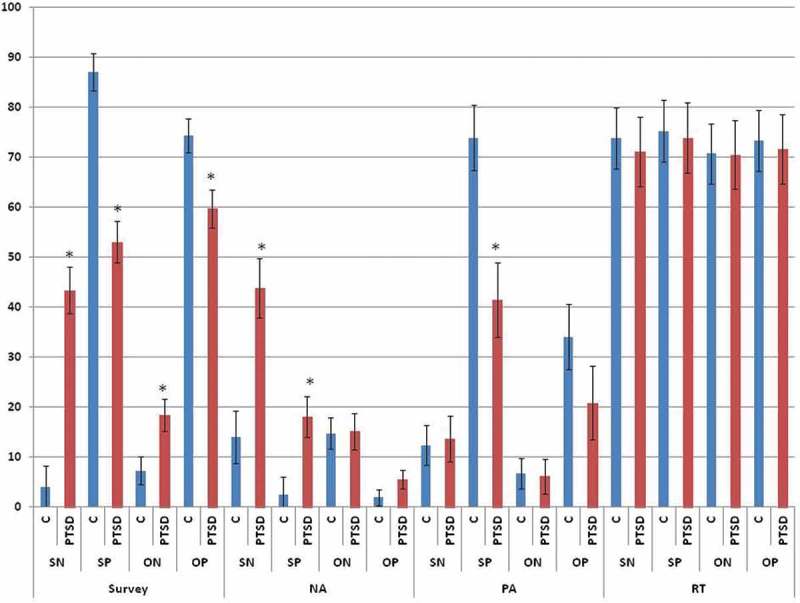
Group differences in self-report and behavioural response to the VV-SORP-T. C = Controls, PTSD = Posttraumatic Stress Disorder, SN = Self-Negative, SP = Self-Positive, ON = Other-Negative, OP = Other-Positive, NA = Negative Affect, PA = Positive Affect, RT = Reaction Time. * group difference at *p* < .05. In the case of survey response, the y-axis is the sum across 10 words referring to how well each word described how participants think about themselves or others, as indicated, on an 11-point scale (0–10) with (0) referring to ‘Not at all’, (5) being ‘Moderately’, and (10) being ‘Completely’. In the case of NA and PA, the y-axis indicates participants’ mean rating on a percentage rating scale (0% referring to ‘Not at all’, 50% indicating ‘Moderately’, and 100% indicating ‘Strongly’) referring to how much they felt ‘anger’, ‘sadness’, ‘anxiety-fear’, ‘disgust’, and ‘bad about self’, in the case of NA, and ‘happy’ and ‘good about self’, in the case of PA. Finally, in the case of RT, the y-axis refers to the obtained response time in msec multiplied by 0.10 in order to conform to the 0–100 scale fitting of the self-report measures.

Figure 2 displays self-report and behavioural responses to the VV-SORP-T by group. Significant multivariate effects were found for *Group, F*(4,34) = 3.004, *p* = .032, *η*2-partial = .261; *Reference, F*(4,34) = 15.571, *p* < .001, *η*2-partial = .647; *Reference by Group, F*(4,34) = 4.106, *p* = .008, *η*2-partial = .326; *Valence, F*(4,34) = 55.492, *p* < .001, *η*2-partial = .867; *Valence by Group, F*(4,34) = 11.156, *p* < .001, *η*2-partial = .568; *Reference by Valence, F*(4,34) = 8.368, *p* < .001, *η*2-partial = .496; and the three-way interaction, *F*(4,34) = 9.204, *p* < .001, *η*2-partial = .520. Follow-up univariate tests of the three-way interaction remained significant after Greenhouse-Geisser correction in the case of survey endorsements, *F*(1,37) = 21.211, *p* < .001, *η*2-partial = .364; negative affect, *F*(1,37) = 8.999, *p* = .005, *η*2-partial = .196; and positive affect, *F*(1,37) = 4.190, *p* = .048, *η*2-partial = .102; but not in the case of reaction time, *F*(1,37) = 0.834, *p* = .367, *η*2-partial = .022.

Referring to survey endorsements, follow-up *t*-tests indicated that, relative to controls and consistent with predictions, PTSD participants reported negative words were more descriptive both of themselves, *t*(38) = 5.556, *p* < .001, *d* = 1.895, and of others, *t*(38) = 2.148, *p* = .041, *d* = 0.707, whereas they reported positive words were less descriptive both of themselves, *t*(38) = 5.564, *p* < .001, *d* = 1.873, and of others, *t*(38) = 2.972, *p* = .006, *d* = 0.960. PTSD participants reported greater negative affect than controls only during self-negative trials, *t*(37) = 3.562, *p* = .002, *d* = 1.184, and during self-positive trials, *t*(37) = 2.580, *p* = .018, *d* = 0.873. Finally, PTSD participants reported less positive affect than controls specifically during self-positive trials, *t*(37) = 3.083, *p* = .005, *d* = 1.025. By contrast, as noted, reaction times did not vary significantly by group as a main effect or in interaction with *Reference* or *Valence*. Instead, only main effects of *Reference, F*(1,37) = 3.043, *p* = .089, *η*2-partial = .076, and *Valence, F*(1,37) = 3.451, *p* < .001, *η*2-partial = .085, were observed, whereby, across groups, reaction times trended toward being slower for SRP than for ORP trials, and for positive than for negative trials.

### 2.2 Neuroimaging results

#### 2.2.1 Components identification

**Figure 3.**
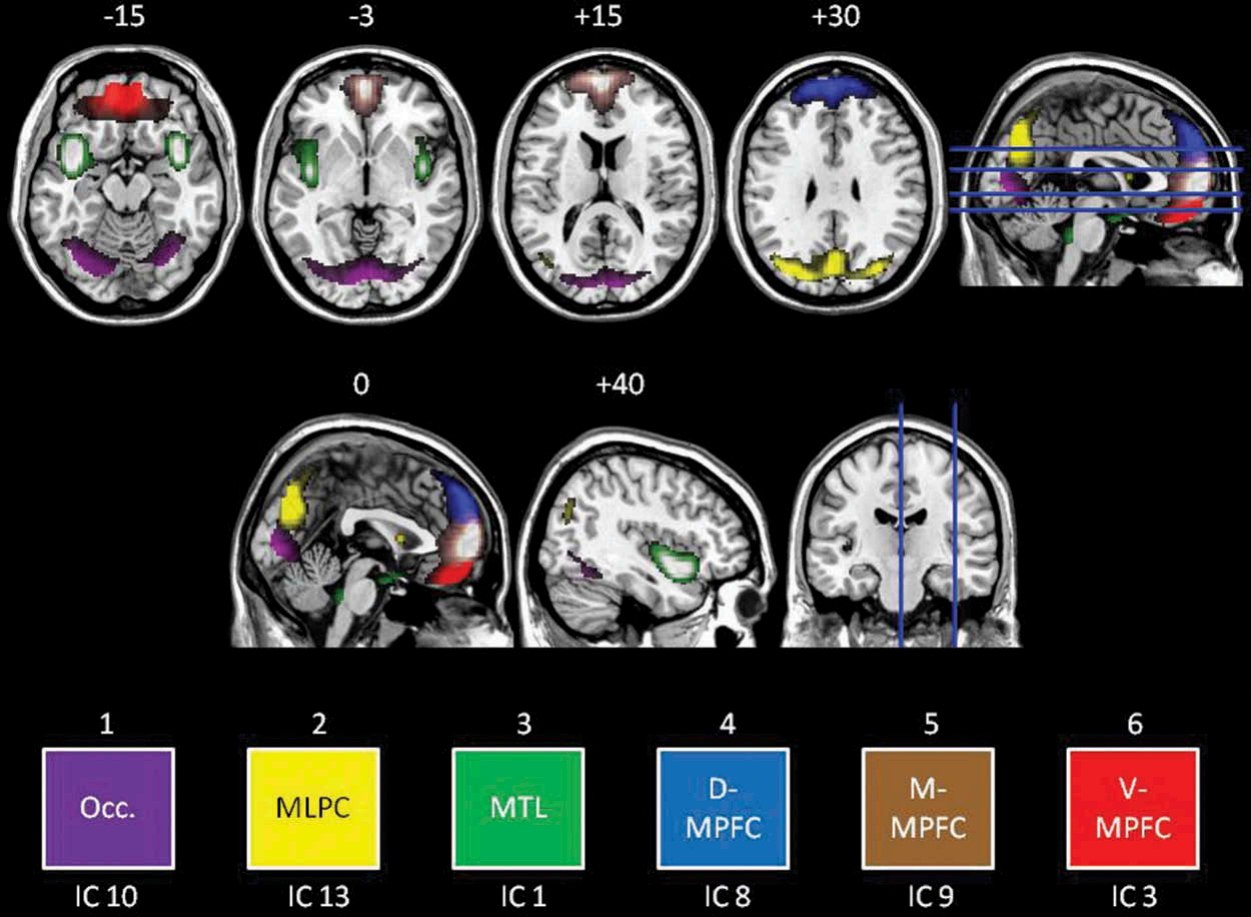
Coloured visualization of independent components. Transverse slices (top row) shown at −15, −3, +15, and +30 mm as per the axial slice. Axial slices (middle row) shown at 0 and +40 (right hemisphere) as per the coronal slice. Six independent components were analysed for group differences as follows: Occ. = Occipital cortex (purple), MLPC = mediolateral parietal cortex (yellow), MTL = medial temporal lobe (green), D-MPFC = dorsal medial prefrontal cortex (blue), M-MPFC = middle medial prefrontal cortex (brown), V-MPFC = ventral medial prefrontal cortex (red).

Six, artefact-free IC were identified, revealing moderate (*r^2^* = .587) to high (*r^2^ = *.683) task-relatedness. A detailed description of each IC is included in the Supplemental data. In summary, we subsequently describe results for six ICs (see Figure 3 for a composite view of the six ICs). Referring to the colours in Figure 3, these ICs were titled: (1) an occipital network (IC 10, *Purple*); (2) mediolateral parietal cortex (i.e. posterior default-mode network; IC 13, *Yellow*); (3) a medial temporal lobe network (MTL; IC 1; *Green*); (4) the dorsal medial prefrontal cortex (MPFC) (D-MPFC; IC 8, *Blue*); (5) the middle MPFC (M-MPFC; IC 9, *Brown*); and (6) the ventral MPFC (V-MPFC; IC 3, *Red*). There was no IC identified that clearly corresponded to the salience network.

#### 2.2.2 Temporal comparisons

Significant differences in the task-relatedness of four of the six components of interest emerged (see Table 2 for *t* and *p* values); again in reference to the colouring of Figure 3, no significant differences were observed for the MTL (green) or D-MPFC (blue) components.

**Table 2.** Significant differences in the neural activity of the ICs in relation to the tasks, in PTSD as compared to controls.

	1	3	8	9	10	13
	MTL	V-MPFC	D-MPFC	M-MPFC	Visual	Posterior DMN
IC	*t (p)*	*t (p)*	*t (p)*	*t (p)*	*t (p)*	*t (p)*
SELF	ns	ns	ns	ns	3.925 (< 0.001)	ns
SP	–				2.692 (0.007)	
SN	–				3.007 (0.003)	
OTHER	ns	2.144 (0.032)	ns	2.649 (0.008)	−4.019 (<0.001)	−2.435 (0.015)
OP	–	3.022 (0.003)		ns	−2.484 (0.013)	ns
ON	–	ns		2.337 (0.02)	−3.228 (0.001)	−2.004 (0.045)

**Legend**: DMN = default mode network, D-MPFC = dorsal medial prefrontal cortex, IC = independent component,

M-MPFC = middle medial prefrontal cortex, MTL = medial temporal lobe, ON = Other negative, OP = Other positive,

SN = Self negative, SP = Self positive, V-MPFC = ventral medial prefrontal cortex.

#### 2.2.3 Self-referential processing

Referring to Figure 3 and Table 2, the PTSD group demonstrated increased activity during SRP within the occipital network (*Purple*; during both self-negative and self-positive conditions).

#### 2.2.4 Other-referential processing

Referring to Figure 3 and Table 2, the PTSD group demonstrated significantly increased activity within V-MPFC (*Red*; specifically during the other-positive condition) and M-MPFC (*Brown*; specifically during the other-negative condition) as compared to women without PTSD. By contrast, the PTSD group demonstrated significantly decreased activity during ORP within occipital cortex (*Purple*; during both other-positive and other-negative conditions), and in mediolateral parietal cortex (*Yellow*; specifically during the other-negative condition) as compared to women without PTSD.

## 3. Discussion

To our knowledge, this is the first neuroimaging study to compare SRP with ORP between persons with vs. without PTSD, consistent with the revised emphasis of PTSD as a disorder of negative alterations in self- and other-referential cognitions and mood under DSM-5 (APA, 2013; Friedman et al., 2011). In agreement with a prior study that investigated visual and verbal SRP alone (Frewen et al., 2011), as compared to women without PTSD, women with PTSD reported more self-descriptiveness of negative words and less self-descriptiveness of positive words, while experiencing more negative affect during both negative- and positive-valenced SRP, and lesser positive affect during positive-valenced SRP specifically. Interestingly, as compared to women without PTSD, women with PTSD also reported negative words were more descriptive of others, while positive words were less descriptive of others, although they did not report experiencing more affective disturbance during ORP. Coinciding with subjective effects, women with PTSD exhibited either increased or decreased response relative to controls in visual cortex, V-MPFC, M-MPFC, and mediolateral parietal cortex (posterior default-mode network), dependent on experimental conditions; these findings will be considered in turn.

Among the most striking study findings included a double dissociation in response within visual cortex during SRP vs. ORP in women with PTSD as compared to controls. Specifically, independent of valence, a pronounced visual cortical response occurred within women with PTSD during trials involving the self (SRP), but a decreased visual cortical response occurred during trials involving others (ORP). Such a response pattern is clearly inconsistent with the presence of a simple visual processing deficit for valenced stimuli generally (e.g. Mueller-Pfeiffer et al., 2013). Instead, collectively these findings suggest that the visual system is activated more so in women with PTSD when having to consider themselves in an emotional context, whilst being shut-down when other people are presented as positive or negative. These responses may be best understood in light of the fact that self-reported affective responses were also stronger for PTSD participants only in response to SRP. Whereas ORP was considered comparably affectively neutral, responding to the self, which tended to prompt an experience of negative affect independent of whether SRP trials were of positive or negative valence, appears to have been associated with increased visual processing of the self face.

In contrast, group differences in response within anterior and posterior cortical midline structures were more apparent in response to trials explicitly involving others (ORP) than those involving the participants themselves (SRP). In the present study, response within MPFC was functionally segregated into three hubs along an inferior-to-superior axis (Ventral [V-MPFC], Middle [M-MPFC], and Dorsal [D-MPFC]). Such findings are important in so far as, within the MPFC, SRP vs. ORP has been more strongly associated with response within V-MPFC vs. D-MPFC, respectively (Denny et al., 2012; Murray et al., 2012). This is, however, directly the opposite of the pattern of findings differentiating the PTSD group from controls observed here. Indeed, whereas in the present study group differences were not observed within the D-MPFC during ORP, women with PTSD demonstrated elevated V-MPFC activity specifically when viewing others in a context of positive association. These findings are interesting given that the V-MPFC is not only strongly associated with reward circuitry (e.g. Bartra, McGuire, & Kable, 2013) but also the affective salience of experiences of self-relatedness and feelings of ‘mineness’ (De Greck et al., 2008; Enzi et al., 2009; Heinzel & Northoff, 2014; Kim & Johnson, 2015; Northoff & Hayes, 2011; Roy, Shohamy, & Wager, 2012). As such, it is interesting to note that whereas several women with PTSD in this study were unable to accept positive associations in reference to themselves due to negative affective interference (e.g. ‘I questioned it, didn’t believe it’; Frewen, Dean, & Lanius, 2012; Frewen, Dozois, & Lanius, 2012; Frewen & Lanius, 2015, p. 115), they were more able to do so in reference to others (e.g. ‘I can accept for others, just not for me’, ‘Much easier, not upsetting’; Frewen & Lanius, 2015, p. 115).

In comparison, during responding to others in the context of negativity, women with PTSD exhibited increased response within M-MPFC coupled with decreased response within the mediolateral parietal cortex, demonstrating a further segregation of the classical default-mode network into components, here anterior vs. posterior, respectively. This pattern of findings may be suggestive of a dissociation between an anterior-mediated narrative (verbal) and posterior-mediated perceptual (visual, embodied) processing of another person presented under the pretext of negative valence. Araujo et al. (2013) also reviewed literature suggesting that the anterior hubs of the default-mode network are more activated by SRP whereas the posterior hubs are more activated by ORP. The latter account also suggests the intriguing possibility that traumatized persons may attribute another person’s negativity self-referentially, due to problems with self-other differentiation and emotional contagion (Hatfield, Cacioppo, & Rapson, 1994; see Nietlisbach, Maercker, Rössler, & Haker, 2010; Parlar, Frewen, Oremus, Lanius, & McKinnon, 2016). Indeed regions of the default-mode network including the MPFC are known to play a role in theory of mind, the ability to attribute the mental states of others (e.g. Völlm et al., 2006). It is possible that the activation differences observed reflect empathetic responses during ORP on the part of PTSD participants, for example, in response to trials associating an unknown person with negativity. Indeed some of the PTSD participants commented that in response to negative ORP that the negative associations were ‘probably not true, I felt bad for her’ and caused them to feel that they ‘wanted to confront her’ (Frewen & Lanius, 2015, p. 115). These other-referenced associations may also produce related self-reflection referring to self-negativity, engaging MPFC circuitry dually involved in theory of mind and autobiographical memory. It will be important to assess the capacity for emotional self-other differentiation in PTSD in future studies.

Findings of the present research must be interpreted in light of study limitations, which in turn are suggestive of future research directions. Generalizability is appropriately limited only to women, and the groups were not equated in age (nor conceivably in socioeconomic status or education, which were not carefully documented). In addition, sample size was not large, and therefore additional group differences may be present but missed due to low statistical power. Moreover, future research should examine the effects of the dissociative subtype of PTSD on SRP and ORP, where Ketay, Hamilton, Haas, and Simeon (2014) found increased M-MPFC and perigenual anterior cingulate cortex response during visual self-face recognition in persons with depersonalization disorder. Whereas the VV-SORP-T integrates visual and verbal processing in a block design, researchers may wish to examine visual and verbal processing independently in traumatized persons, before vs. after therapy and recovery. Indeed whether SRP and ORP tasks involve principally visual vs. verbal stimulus processing is likely relevant to the interpretation of findings; for example, whereas an emotional picture viewing task produced decreased D-MPFC and ACC activity in depressed persons in response to negatively-valenced pictures that were judged to be self-relevant (Grimm et al., 2009), in exclusively verbal SRP tasks, depressed persons have rather shown increased M-MPFC and ACC response (Lemogne et al., 2009; Li et al., 2017; Yoshimura et al., 2010). A limitation common to these and other prior studies of SRP and ORP in psychiatric and/or vulnerable samples, however, includes a lack of measurement of psychological trauma history, which may be a non-specific etiological factor for various disturbances in SRP and ORP associated with psychopathology. Future studies should therefore compare SRP and ORP in persons with different disorders (e.g. PTSD vs. MDD), as a function of the presence vs. absence of significant trauma histories; a limitation of the present study is that we did not include a psychiatric control group and therefore cannot attribute group-level findings solely to the presence of PTSD. Further, differences in reaction time for SRP and ORP as potentially indicating differential depth of reflective processing during SRP (Frewen & Lundberg, 2012) potentially reflect a confound for interpreting neural effects (Yaoi, Osaka, & Osaka, 2013).

Currently some evidence-based psychotherapies for PTSD have as a focus cognitive restructuring interventions designed to lessen negative SRP, where the general principles of cognitive therapy alone, without trauma memory processing, may sufficiently treat PTSD in certain cases (Resick et al., 2008). The present findings may also be clinically significant in so far as it has been argued that a greater understanding of the intrinsic connectivity networks involved in disturbances in SRP and ORP in traumatized persons may ultimately support the design of neuroscientifically-informed treatments for PTSD (e.g. Lanius, Bluhm, & Frewen, 2011; Lanius, Frewen, Tursich, Jetly, & McKinnon, 2015). For example, studies like the present one highlight regions of interest for neurofeedback and neurostimulation treatments.

In summary, to our knowledge, this is the first neuroimaging study to investigate valenced SRP and ORP in persons with vs. without PTSD. In comparison with controls, women with PTSD reported negative words were more descriptive of both themselves and others, the opposite being true of positive words. Women with PTSD also reported a greater experience of negative affect during both negative- and positive-valenced SRP, and a diminished experience of positive affect in response to positive-valenced SRP. Clinicians and researchers should attend to negative SRP and ORP in traumatized persons, as this may present in self-conscious, internally-directed emotions such as posttraumatic guilt and shame. Finally, a number of functional brain-based differences were observed during SRP and ORP within visual cortex and cortical midline structures associated with the default mode network. We suspect that repetitive trauma exposure, including childhood abuse and neglect, may engender the development of brain-based disturbances in SRP and ORP that present across the spectrum of trauma-related psychological disorders. A better understanding of such processes through neurophenomenology will hopefully culminate in novel treatments and improved opportunities for trauma recovery.

## Supplementary Material

Supplementary material

## Highlights

- First neuroimaging comparing valenced Self-referential Processing (SRP) to Other-Referential Processing (ORP) in women with versus without PTSD.
- Women with PTSD endorsed negative words as more descriptive both of themselves and others and reported greater negative affect and lesser positive affect.
- Significant differences between groups were observed within Cortical Midline Structures and Occipital Cortex during both SRP and ORP.
- Treatment should address altered sense of self and response to others in PTSD.
