# Supplementary material for: Neuroimaging the traumatized self: fMRI reveals altered response in cortical midline structures and occipital cortex during visual and verbal self- and other-referential processing in women with PTSD
Source: Eur J Psychotraumatol. 2017 May 16;8(1):1314164. doi: 10.1080/20008198.2017.1314164 (PMC5475303; doi:10.1080/20008198.2017.1314164)
Supplement: Supplementary material [file zept_a_1314164_sm6701.docx]

**Supplementary Materials**

**Functional MRI**

**fMRI data acquisition**

Brain images were acquired using a 3.0 Tesla whole-body MRI scanner (Magnetom Tim Trio, Siemens Medical Solutions, Erlangen, Germany) with a 32-channel phased array head coil. T1-weighted anatomical images of the whole head were collected with 1mm isotropic resolution (MP-RAGE, TR/TE/TI = 2300ms/2.98ms/900ms, flip angle = 9 degrees, FOV = 256mm x 240mm x192mm, acceleration factor = 4, total acquisition time = 3min 12s). Functional images were acquired using a gradient echo planar imaging (EPI) sequence with an interleaved slice acquisition order and tri-dimensional Prospective Acquisition Correction (3D-PACE). EPI volumes were acquired with 2mm isotropic resolution and the following parameters: FOV=192mm x 192mm, 94 x 94 matrix, TR/TE = 3000ms/20ms, flip angle = 90 degree, 64 slices, 178 measurements.

**Preprocessing of imaging data**

*Statistical Parametric Mapping 8* (SPM8, Wellcome Trust Centre for Neuroimaging, London, UK) implemented in *MATLAB R2013a* (Mathworks Inc., Sherborn, MA, USA) was used to preprocess functional and structural acquired images. Individual functional images were corrected for motion by realignment to the first volume of each session. All images were spatially normalized (2 × 2 × 2 mm) to an EPI in MNI space and spatially smoothed using a full width at half maximum (FWHM) isotropic Gaussian kernel of 8 mm.

**Components Identification**

Six, artifact-free independent components (IC) were identified that revealed moderate (*r^2^* = .587) to high (*r^2^ =*.683) relatedness with task conditions. A detailed description of each IC is included in the supplementary materials. The first IC (i.e., IC 1) encompassed the bilateral insula, superior temporal pole, the left parahippocampal gyrus, hippocampus and amygdala, and the left inferior frontal cortex. Although this IC spatially correlated strongly with the salience network (*r* = .754, Ros et al., 2013), noticeably absent from this medial-temporal IC in respect of its mapping with the salience network was its exclusion of the dorsal anterior-mid cingulum, a key hub for the salience network; we therefore restrict our interpretation of this IC to the medial-temporal lobe (MTL). Three independent components primarily encompassed the medial prefrontal cortex (MPFC) as differentiated along an inferior-superior axis (ICs 3, 8, and 9). Specifically, the first (IC 3) included bilateral ventral prefrontal regions (ventromedial prefrontal, medial prefrontal, and middle frontal cortex [Brodmann areas, BA10,11], and ventral anterior cingulum [BA 25, 32]), and was therefore considered the ventral MPFC (V-MPFC). The second (IC 9) included the bilateral MPFC (from the V-MPFC [dorsal BA 11] up to BA 9 and comprising the rostral anterior cingulum [BA 24, 32]) and was significantly spatially correlated with the dorsal default mode network (*r* = .569, Shirer et al., 2012); this IC was labelled the middle MPFC (M-MPFC) accordingly. The third (IC 8) mainly comprised the bilateral medial superior frontal cortex (BA 8, 9, 10) and was thus labelled the dorsal MPFC (D-MPFC). Another component (IC 13) included parieto-occipital regions, comprising the bilateral precuneus and the superior regions of the bilateral cuneus (BA 7, 18, 31), extending to the posterior middle temporal gyri; this component thus included the posterior regions of the default-mode network and was indeed found to correlate with a standard mask of this network (*r* = .635, Garrity et al., 2007). Finally, IC 10 mainly encompassed the visual cortex (bilateral cuneus, lingual and fusiform gyri [BA 17, 18, 19]) but also included the posterior cerebellum (lobule 6); this IC was found to be spatially correlated with the primary visual network (*r* = .249; Shirer, Ryali, Rykhlevskaia, Menon, & Greicius, 2012).
